# Prolactin: The Third Hormone in Breast Cancer

**DOI:** 10.3389/fendo.2022.910978

**Published:** 2022-06-16

**Authors:** Linda A. Schuler, Kathleen A. O’Leary

**Affiliations:** Department of Comparative Biosciences, School of Veterinary Medicine, University of Wisconsin-Madison, Madison, WI, United States

**Keywords:** prolactin (PRL), breast cancer, mammary cancer, luminal breast cancer, HER2+ breast cancer, STAT 5 transcription factor, triple negative breast cancer

## Abstract

Prolactin coordinates with the ovarian steroids to orchestrate mammary development and lactation, culminating in nourishment and an increasingly appreciated array of other benefits for neonates. Its central activities in mammary epithelial growth and differentiation suggest that it plays a role(s) in breast cancer, but it has been challenging to identify its contributions, essential for incorporation into prevention and treatment approaches. Large prospective epidemiologic studies have linked higher prolactin exposure to increased risk, particularly for ER+ breast cancer in postmenopausal women. However, it has been more difficult to determine its actions and clinical consequences in established tumors. Here we review experimental data implicating multiple mechanisms by which prolactin may increase the risk of breast cancer. We then consider the evidence for role(s) of prolactin and its downstream signaling cascades in disease progression and treatment responses, and discuss how new approaches are beginning to illuminate the biology behind the seemingly conflicting epidemiologic and experimental studies of prolactin actions across diverse breast cancers.

## 1 Introduction

Factors that regulate cell-specific proliferation and differentiation repeatedly have been shown to be significant actors in oncogenesis and potential therapeutic targets in established cancers. Prolactin (PRL) cooperates with the ovarian steroids, estrogen and progesterone, to orchestrate the cycles of mammary development and differentiation that lead to successful lactation, providing nourishment for the offspring. PRL-initiated signals that expand alveolar cells during pregnancy and coordinate their differentiation at the time of birth have been mechanistically defined [ (1–4) and references therein]. The essential actions of PRL in these physiological processes have suggested roles in breast cancer [ (5–14) and references therein], by analogy to the recognized roles of the two other major hormones that regulate mammary development and function, estrogen and progesterone. Yet understanding its activities and consequences across diverse clinical breast cancers in order to develop prevention or treatment strategies has been elusive.

While control of PRL expression by pituitary lactotrophs during pregnancy and lactation is well understood [reviewed in (15)], its expression outside of pregnancy has received less attention. Pituitary PRL secretion is influenced by many factors, and circulating levels in nonpregnant women vary considerably (16–18). In addition to physiological stimuli, estrogen-progestin menopausal hormone therapy (MHT) raises circulating PRL (18), and anti-psychotics that antagonize dopamine induce hyperprolactinemia (19, 20). Further, PRL also can be expressed by non-lactotrophs, including within the mammary gland (21–23), and by breast cancer cells themselves (24–27). COX-2 (PTGS2) can induce PRL expression in fibroblasts, including at potential metastatic sites, mediated by PGE2 induction of NR4A (28). Moreover, in contrast to growth hormone (GH) in nonprimates, hGH is also a potent PRL receptor agonist (29, 30). Like PRL, it can be produced locally by breast cancer cells (26), and hGH and PRL receptors can heterodimerize (31). Thus, PRL receptors (PRLR) in the breast may be exposed to agonists from local and circulating systemic sources, even in the absence of pregnancy.

Here we review the epidemiologic evidence linking PRL to oncogenesis in the breast, and recent experimental studies implicating multiple underlying mechanisms. We then address the more controversial role(s) for PRL in established breast cancers. PRLR is highly expressed in many breast cancers across all different subtypes, and epidemiologic analyses and experimental studies are revealing that PRL can elicit both pro-differentiation and pro-aggression outcomes. We discuss how new approaches are illuminating the factors that determine the responses to PRL, including intrinsic tumor cell properties and the microenvironment, and point to directions for future studies that will integrate our understanding of this hormone in breast cancer progression and therapeutic responses.

## 2 PRL Actions in Development of Breast Cancer

### 2.1 Epidemiological Studies

Multiple epidemiologic studies have examined the relationship between levels of circulating PRL and development of breast cancer [meta-analysis and review (32),]. Large prospective studies have linked higher levels of circulating PRL within the normal range to increased risk for breast cancers which express estrogen receptor alpha (ER+) in postmenopausal (16, 33), or premenopausal women (34). In the study nested within the Nurses’ Health Study, PRL levels predicted breast cancer risk independent of estrogen (35). Additional analyses of this cohort found that the association of circulating PRL in the highest quartile in postmenopausal women ten years prior to diagnosis was strongest for aggressive ER+ breast cancer (36). Furthermore, epidemiologic studies have linked PRL to mammographic density (34, 37, 38), a potent independent contributor to increased breast cancer risk (39, 40). Incorporation of PRL in risk prediction models improves their efficacy (34, 41). Conversely, the reduced PRL levels in parous compared to nulliparous women may play a role in the long term protection conferred by pregnancy (16, 18, 34, 42).

### 2.2 Experimental Studies

#### 2.2.1 *In Vivo* Models

The ability of PRL to stimulate mammary tumorigenesis in rodent models has been recognized for some time. Many early studies manipulated pituitary PRL (5–7), especially using pituitary isografts transplanted to the kidney capsule to chronically elevate circulating PRL by removing the inhibitory effects of dopamine (43). This approach reveals effects of PRL in combination with progesterone; in rodents, PRL also supports the corpus luteum (44).

More recently, genetically modified mice have permitted interrogation of mechanisms by which PRL may increase risk of breast cancer, apart from ovarian steroids. Transgenic PRL under the control of several promoters leads to mammary cancers [reviewed in (45)], as does transgenic mammary STAT5A, the canonical mediator of PRL signals (46). PRL drives development of mammary cancers in mice with germline ablation of *Stat1* secondary to somatic truncating mutations in *Prlr*, resulting in an alternatively spliced protein resembling the human “intermediate” isoform (47) (see Sections 3.1, 3.4.2). Our group generated the NRL-PRL mouse (48, 49), in which transgenic rat PRL is expressed by mammary epithelia, mimicking the local PRL synthesis in breasts of women (23). Unlike circulating PRL, this locally elevated PRL does not disturb estrous cycling, enabling study of the interactions of PRL with ovarian hormones, of particular importance when assessing models of pre- and post-menopausal breast cancer. Mammary glands of young adult NRL-PRL females exhibit elevated pERK1/2 and pAKT, in addition to pSTAT5 (50), reflecting the spectrum of PRL-initiated signaling cascades (22, 51). These mammary glands exhibit both ductal abnormalities (mammary intraepithelial neoplasias, resembling ductal carcinoma in situ, DCIS), and epithelial hyperplasias (48, 52). With age, nulliparous females spontaneously develop histologically diverse, metastatic ER+ carcinomas with long latencies, mirroring the epidemiologic link between PRL exposure and aggressive ER+ cancer (36). These tumors can develop without postpubertal ovarian steroids, similar to the observation that the increased risk conferred by PRL in women is independent from estrogen (16), although supplemental 17β-estradiol decreases tumor latency (50). Once established, tumors are no longer dependent on estrogen for growth, modeling clinical anti-estrogen resistant luminal B cancers (53–55). However, the ER remains functional; estrogen activity modulates tumor gene expression and behavior, including proliferation and cancer stem cell activity (54, 56).

In order to understand the molecular events underlying PRL-driven oncogenesis, we performed comprehensive genomic profiling over the course of disease (57). Similar to clinical ER+ breast cancers (58–60), end stage tumors exhibited few nonsynonymous somatic mutations. However, nearly 80% of tumors showed alterations in the Ras pathway, including canonical activating mutations and copy number amplifications of *Kras*. Interestingly, many aggressive clinical ER+ breast cancers exhibit elevated Ras pathway activity as a result of mutations in the Ras proteins, or reduced expression or somatic loss of Ras-GAP tumor suppressors (61–64). Many of the remaining 20% of experimental PRL-induced cancers exhibited elevated pAKT, but not pERK1/2, consistent with driver mutations in the phosphatidylinositol-3-kinase pathway, common in many clinical ER+ cancers (63, 65, 66). Transcriptomic analyses showed that tumors expressed variable transgenic PRL compared to preneoplastic tissue, suggesting divergent PRL influence once tumors are established. These analyses also revealed marked alterations in cell-intrinsic processes and the tumor microenvironment, including immune activity. Consistent with low numbers of intratumoral lymphocytes, including CD8+ effector T cells, but large numbers of infiltrating macrophages, tumors contained strikingly reduced transcripts for many chemokines and indicators of anti-tumor immunity. This immunosuppressed environment resembles that of clinical ER+ breast cancers [reviewed in (67, 68)].

#### 2.2.2 Direct Actions on Mammary Epithelia

**Figure 1:**
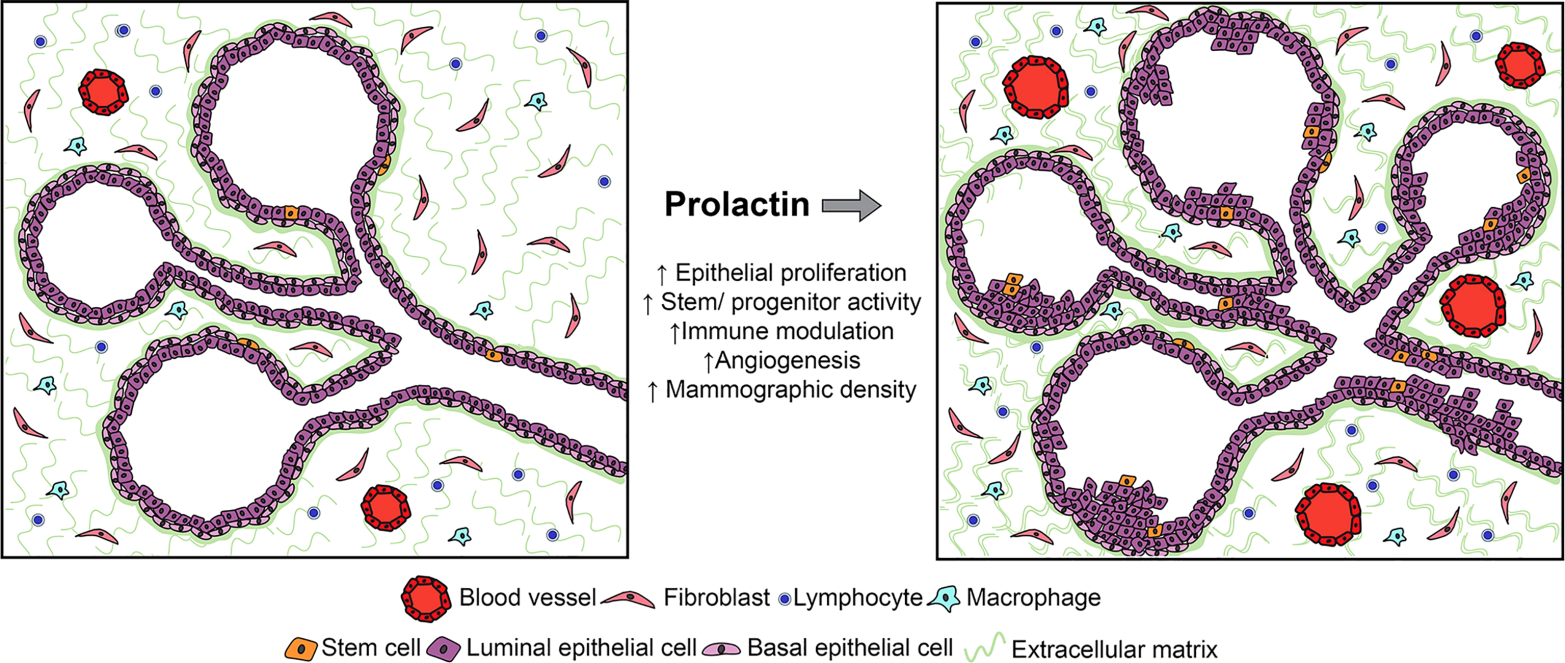
Studies of human samples and experimental models have shown that PRL can act on multiple target cells within the mammary environment, including not only epithelia, but also stromal cells, including immune and fibroblastic cell subpopulations. Although its effects on epithelia are best understood in the context of breast cancer, its actions on stromal targets which have been defined in other systems would also be predicted to increase the risk for breast cancer. See Section 2.2 for details.

Extensive studies of the direct actions of PRL on breast cancer cells *in vitro* have demonstrated increased proliferation and cell turnover [reviewed in (22)], and these effects are also observed in normal mammary epithelia in the dynamic *in vivo* environment in multiple murine models (2, 45, 49). In addition, recent studies have revealed that PRL powerfully influences the mammary epithelial hierarchy, both independent of ovarian steroids and in concert with these hormones (69) (**Figure 1**).

In the NRL-PRL model, local PRL increased epithelial stem/progenitor activity and dampened the regulatory networks which drive differentiation (69). In ovariectomized young adult females, transgenic PRL increased luminal progenitors; in combination with estrogen and progesterone, PRL increased bilineage progenitors, and raised stem cell activity associated with augmented canonical Wnt signaling. However, PRL opposed steroid-driven luminal maturation, associated with reduced *Gata3* and higher *Sox9* transcripts (69). A growing literature supports stem/progenitor cell populations as cancer cells of origin (70), and mammary luminal progenitors have been implicated as precursors for multiple subtypes of breast cancer [reviewed in (71)]. The ability of PRL to expand these epithelial subpopulations would contribute to increased cancer risk.

#### 2.2.3 Effects on Non-Epithelial Cells

Multiple non-epithelial cells in the mammary environment have been reported to express PRLR (72, 73). Although few actions of PRL at stromal targets have been addressed experimentally in the context of breast cancer, data from physiologic and other pathologic states suggest the need for additional study. The critical roles of the immune system in mammary development, lactation and involution are increasingly appreciated (74–76). PRL, like the ovarian steroids, can influence mammary immune cell content and activity, both indirectly by altering epithelial cytokine secretion (77), as well as directly acting on both innate and adaptive immune cell subpopulations (78–83). Studies of lymphocyte activation *in vitro* showed a bimodal concentration-dependent response to PRL (84, 85), suggesting the intriguing possibility that mammary PRL synthesis may influence local immune activity. Together, these reports suggest that PRL may modulate inflammation and/or immunotolerance during tumorigenesis, with potential to contribute to a permissive environment for development of breast cancer.

PRL-induced synthesis of components of the extracellular matrix (ECM) by both epithelial and non-epithelial target cells may complement mitogenic effects of PRL on mammary epithelium to augment mammographic density, suggesting another mechanism by which PRL may raise risk (86). In this regard, the ability of PRL to stimulate macrophages to augment fibrosis in pancreatic cancer is of interest (87). Furthermore, PRL can modulate angiogenesis. As an intact protein, it can promote vascularization (88, 89); in contrast, its proteolytic products (vasoinhibins) impede this process (90). These activities have suggested roles in normal mammary function as well as breast cancer (91).

#### 2.2.4 Contributions to Growth of Early Lesions

These actions of PRL on breast epithelia and potentially on other stromal cells could support development of breast cancers. Moreover, these activities would fuel early lesions regardless of the initiating event (**Figure 1**). Many clinical DCIS lesions express PRLR (92), and the PRL antagonist, Δ1-9-G129R-hPRL, inhibited the mammosphere-forming activity of primary DCIS samples (93). The rich literature elevating systemic PRL using pituitary isografts in mouse models demonstrates that PRL in combination with progesterone can promote carcinogen- and p53 null-induced tumors [e.g., (6,94)]. Conversely, germline genetic ablation of *Prl* or *Prlr* slowed growth of lesions induced by viral oncogenes (95, 96). Antipsychotics that act by antagonizing dopamine, thereby raising circulating PRL, promoted tumorigenesis in experimental models initiated by RCAS-caErbB2, RCAS-HrasQ61L and MMTV-Wnt-1 (97). A recent study found that patients using these drugs had a significantly increased risk of breast cancer (20). Together, these observations in patients and murine models indicate a role(s) for PRL in progression of early lesions.

#### 2.2.5 Cooperation With Other Factors

##### 2.2.5.1 Estrogen, Progesterone

In patients, PRL would act in concert with other hormones and potentially carcinogenic factors, as well as dysregulation of multiple pathways as disease progressed. Prior to menopause, PRL would interact with ovarian steroids; after menopause, estrogen/progestin MHT would continue this crosstalk. In the European Prospective Investigation into Cancer and Nutrition cohort, postmenopausal women with higher circulating PRL who had used combined estrogen/progestin MHT had the most significant increase in incidence of ER+ breast cancer (33). Estrogen in the absence of progestins also would be an actor in postmenopausal women receiving either estrogen only MHT, or in untreated women by extraovarian estrogen synthesis (98, 99). PRL cooperates with estrogen, a well-recognized risk factor for breast cancer, by multiple mechanisms, including reciprocal upregulation of the other’s receptors (100, 101), and downstream crosstalk (102, 103). Supplemental estrogen accelerates PRL-driven mammary cancers in the NRL-PRL model (50). Furthermore, PRL induced pAKT and pERK1/2 can activate ERα in the absence of estrogen ligand *in vivo* as well as *in vitro* (104–106). PRL interaction with progesterone has been best studied in the context of pregnancy and lactation, where these hormones cooperatively drive expansion of alveolar cells during pregnancy, but oppose one another to initiate lactation (3). Outside of pregnancy, they regulate the other’s receptors, and as observed above, work together to increase mammary stem cells (69, 107, 108).

##### 2.2.5.2 Other Oncogenic Factors

Locally elevated transgenic PRL also has revealed potent collaboration with other oncogenic pathways. Crosses between NRL-PRL mice and other murine models of mammary cancer, including elevated TGFα, loss of p53, and mutagen with increased canonical Wnt signals conferred by an inactivating mutation in the tumor suppressor APC, dramatically reduced tumor latency or increased tumor incidence (52, 104, 109–111). Transgenic PRL not only enhanced carcinogenesis, but also markedly influenced the resulting cancers in ways that would impact treatment responses. For example, transgenic local PRL increased the proportion of claudin low tumors in the absence of p53 (110), and in the presence of mutated APC, elevated PRL resulted in tumors with Notch-dependent cancer stem cell activity, compared to the β-catenin-dependence observed in tumors with mutant APC alone (111). Further, transgenic PRL and TGFα in combination sustained activation of the ERK1/2 and AKT signaling cascades (104, 109), reflecting the potent cooperation of PRL with growth factor-initiated signals (112, 113). This further activates ERα in the absence of estrogen ligand *in vivo* (104, 106, 109), one mechanism which underlies resistance of ER+ breast cancers to anti-estrogens (114, 115) (Section 3.3 below). In contrast to the positive interactions between PRL and growth factors in these transgenic murine and breast cancer models, PRL and EGF have been reported to oppose one another in “normal” mammary cell lines, such as HC11 and NMuMG; the phenotype of the target cell is likely to be critical in dictating the outcome of PRL signals and crosstalk with other signals (112, 116).

## 3 Role of PRL in Established Breast Cancers

In contrast to the strong epidemiologic data supporting a role for PRL in development of breast cancer, particularly of ER+ tumors, its role in established cancers continues to be actively debated. Much of the discussion revolves around the extent of PRLR expression by the tumor parenchyma, including which breast cancer subtypes and which PRLR isoforms, and importantly, whether PRL fuels tumor aggression or fosters a more differentiated phenotype.

### 3.1 PRL Receptors in Clinical Breast Cancers

PRLR isoforms with distinct intracellular domains and consequent differing signaling capacities are generated by alternative splicing. The full length “long” PRLR isoform is best studied, but as noted below, expression of the “intermediate” PRLR isoform in breast cancers is also recognized. Homo- and hetero-dimerization of these PRLR isoforms not only influences the repertoire of potential signaling pathways, but also stability of the receptors [reviewed in (22, 117)]. These isoforms have further confounded detection of PRLR across breast cancer subtypes, which is already complicated by the specificity and sensitivity of historically available antibodies (117, 118). However, multiple recent studies have reported PRLR protein expression in ER+, HER2+ and triple negative (TNBC) breast cancers, in sharp contrast to the epidemiologic link between PRL and development of only ER+ breast cancers. The relative proportion of tumors within each subtype that expressed PRLR varied with the cohort examined, antibody utilized [i.e., detecting the extracellular domain shared by most PRLR isoforms (119–121), intracellular domain of the full length “long” PRLR (92, 122), or unique intracellular domain of the “intermediate” PRLR isoform (117)], and other methodological differences. Given these variables, it is not surprising that the proportion of PRLR-expressing breast cancers varied from 25-83%.

Although PRLR expression was independent of ER (92, 119, 120, 122), PRLR levels were highest in ER+ tumors when analyzed (92, 123). Some of these studies found highest PRLR expression in more differentiated tumors in patients with longer metastasis free survival (92, 119). In contrast, Shemanko and her colleagues found that higher tumor PRLR protein levels correlated with a shorter time to bone metastasis, consistent with experimental PRL-induced osteoclast differentiation (120). Some of these reports included interrogation of relative transcript abundance and outcomes in various publicly available databases; with the caveat that transcript levels may not reflect protein expression, these results also differed (92, 123).

Although overall, TNBCs expressed less PRLR detected with an antibody to the intracellular domain of the “long” PRLR (92), a subset of these tumors expressed higher PRLR levels. This TNBC subset was found to be more differentiated, supporting the hypothesis that PRL drives a pro-differentiation program in these cancers (122). (*See review by Ali and colleagues elsewhere in this series*). Interestingly, however, Clevenger and colleagues reported that the “intermediate” isoform of the PRLR, which would not have been detected with the antibody used in the study above, was most highly expressed in TNBC (117). Moreover, they found that cancers with a high ratio of transcripts for the “intermediate” to the “long” PRLR isoform were associated with greater likelihood of distant metastases in the TCGA database. Experimentally, heterodimers of these PRLR isoforms were more stable, and less able to activate STAT5 (Section 3.4.2 below). Clinical TNBCs are very heterogeneous (124, 125); together, these reports suggest that different TNBC subsets may respond quite differently to PRL.

Although many of these studies correlated levels of PRLR protein or transcripts with prognosis, albeit with conflicting conclusions, the outcome of PRLR signaling has been directly addressed only in small Phase I/II studies of patients with advanced disease. A study of a PRLR neutralizing antibody as a monotherapy that included 34 breast cancer patients (all subtypes, but 75% ER+ cancers) found no significant effect on disease progression (126). In another small Phase II trial (20 breast cancer patients), the dopamine D2 receptor agonist, cabergoline, was used to inhibit secretion of pituitary PRL; two of these patients experienced extended disease control (127). The lack of definitive positive results dampened enthusiasm, although the small number of patients, the advanced stage of their disease, and extensive pretreatment regimens limit interpretation. However, interest in this area persists. Conjugates of other therapeutic agents to PRL antagonists or PRLR neutralizing antibodies are being developed, as discussed further in Section 3.3 below.

### 3.2 Experimental Studies Using Xenografts

Mouse PRL has little activity at the human PRLR (30), which has complicated experimental study of breast cancers *in vivo*. However, Rui and his colleagues have developed a mouse in which the mouse *Prl* gene has been replaced with the human *PRL* gene (NSG-Pro), resulting in physiologic regulation of hPRL expression (128). In these recipients, ER+ patient derived xenografts (PDXs) displayed a remarkable 15-20 fold higher transplantation rate than in wildtype NOD SCID gamma (NSG) mice. Moreover, the NSG-Pro mice facilitated metastatic dissemination and growth of distant lesions, genetic evolution and development of anti-estrogen resistance (128). These studies support an important role for PRL in the biology of ER+ tumors.

Well-characterized breast cancer cell lines modeling different breast cancer subtypes have been extensively studied *in vitro* to understand the outcomes of PRL actions, and to dissect its signals and mechanisms of interaction with other factors; these reports will not be further reviewed here. (*See reviews by Ali and Clevenger and their colleagues elsewhere in this series*). Some investigators have examined PRL responses in murine recipients of transplanted cell lines by providing another source of hPRL, with conflicting results. Primary tumors of transplanted MDA-MB-468 breast cancer cells that expressed hPRL grew more rapidly than tumors that did not in *nu/nu* (nude) mice (129). Reduction of the “long” PRLR isoform reduced pulmonary and hepatic metastatic burden in NOD-SCID recipients of HER2+ BT474 cells, which were supplemented with hPRL (130). In contrast, hPRL-treatment of NOD/SCID mice bearing xenografts of MDA-MB-453 breast cancer cells reduced tumor growth and dissemination (122), and growth of primary HER2+ SKBR3 tumors (131).

In a different approach, Ali and her colleagues used CRISPR/Cas9 to reduce PRLR expression in the ER+ MCF7 and HER2+ SKBR3 breast cancer cell lines (132). Loss of PRLR in MCF7 cells reduced ER expression, consistent with regulation of *ESR1* by PRL, and promoted less differentiated, more aggressive tumors upon transplantation to NOD/SCID mice. In HER2+ SKBR3 cells, loss of PRLR increased HER2 expression, and ability to colonize lungs of NSG recipients. These findings, together with associated *in vitro* analyses, indicate beneficial actions of PRLR in these models (132). (*See review by Ali and colleagues elsewhere in this series*).

The basis for the disparate responses to PRL in these xenograft studies is unclear. There are many differences among these experiments. The transplanted cancer cells, whether PDXs or different breast cancer cell lines (133, 134), are quite distinct. Moreover, the design of these studies differs markedly, including placement of the transplanted cells, the extent that the murine hosts are immunocompromised, and method of manipulating PRL activity. Additional studies are necessary to understand how these findings reflect diverse clinical breast cancers.

Few studies have been performed in syngeneic experimental models. However, the findings are intriguing. In a murine model of HER2+ cancer (MMTV-neu), the PRL antagonist, G129R-hPRL, reduced metastases after removal of the primary tumor (135). In a subsequent study, the effects of G129R-hPRL on HER2 signaling in this model were found to be dependent on cancer associated fibroblasts (136), supporting the importance of study of complex systems with multiple cell types. Systemically reducing expression of the “long” PRLR isoform using a novel method reduced metastases in the aggressive 4T1 model, and PRL-supported immunosuppressive Tregs were identified as a major target (83, 130). These observations underscore the drawbacks of xenograft models. Most notably, currently available xenograft recipients are severely immunocompromised, lacking critical components of the host response. Ongoing efforts to develop mice with “humanized” immune systems will address this shortcoming. In addition, subtle differences in the structures of mouse/human proteins can obscure paracrine/systemic communication between tumor and stromal cells, e.g., PRL itself (30).

### 3.3 PRL Interactions With Other Treatment Approaches

Although PRL/PRLR inhibitors have not shown robust promise as monotherapies, there has been long term interest in their interaction with other treatment modalities, especially with anti-estrogens in ER+ breast cancers. As noted in Section 2.2.5, PRL cooperates with estrogen by multiple mechanisms, which have been dissected primarily in the well-differentiated ER+ breast cancer cell line, MCF7 (100–103). Not surprisingly, as for other aspects of cancer biology, this relationship evolves with disease progression. In an experimental rat model of hormonally-responsive ER+ mammary cancer, concomitant inhibition of PRL and aromatase cooperatively reduced tumor growth (137). Similarly, in therapy naïve ER+ PDXs transplanted to NSG-Pro recipients, PRL initially supported anti-estrogen responsiveness, but with time, the PRL environment facilitated development of resistance to tamoxifen (128). This was associated with increased growth factor signals, including ligand independent activation of ER, a potent outcome of PRL-growth factor crosstalk (See Section 2.2.5), and activation of the ERBB2 pathway (128). This relationship between PRL and resistance to anti-estrogens is reflected in some but not all small clinical studies [reviewed in (16)]. Interestingly, LAT1/SLC7A5, a transporter for branched chain amino acids which is regulated by PRL during lactation (138), is highly expressed by tamoxifen resistant cancers (139–141). The growing recognition of the importance of tumor metabolism, and role of PRL in regulation of metabolism during lactation, points to this area for further study.

The potent crosstalk of PRL with growth factor-initiated signals observed both in breast cancer cell lines (112, 142), and anti-estrogen resistant ER+ PDXs (128) has suggested that targeting PRL signaling in combination with these pathways may be an efficacious therapeutic strategy. PRL can initiate phosphorylation of HER2 in SKBR3 and BT474 breast cancer cells *in vitro* and in a murine MMTV-neu-derived tumor ex vivo (136, 143, 144). In light of the apparent conflict of these *in vitro* observations with the results of some but not all xenograft studies as noted in Section 3.2, this deserves additional investigation.

Several small clinical studies suggested that reduction of PRL might improve responses to chemotherapies [reviewed in (16, 145)]. The ability of PRL to promote survival of breast cancer cells *in vitro* has been appreciated for some time [reviewed in (22, 145)]. In breast cancer cell lines representing different cancer subtypes, PRL promoted to resistance to chemotherapies, including doxorubicin, paclitaxel, and cisplatin (93, 146, 147). Several related mechanisms have been identified, including PRL-induced expression of anti-apoptotic proteins (148), transcription of the multidrug resistance transporter ABCG2 (149), and activation of glutathione-S-transferase (147). Interaction with chemotherapies has not been directly revisited clinically, but these actions may contribute to the efficacy of compounds conjugated to anti-PRL agents, as noted below.

The relatively low toxicity of PRL antagonists and PRLR neutralizing antibodies and widespread PRLR expression across different breast cancer subtypes have prompted their development as delivery vehicles for other therapeutic agents, including cytotoxic compounds (121, 135, 150, 151), anti-HER2 (152), and immunomodulators to attract and/or activate CD8+ T cells (135, 153). In addition to targeting delivery of other treatments, testing of these molecules will also provide information on the efficacy of concomitant inhibition of PRL signals.

### 3.4 PRL Initiated Signals

#### 3.4.1 Canonical JAK2-STAT5 Pathway

**Figure 2:**
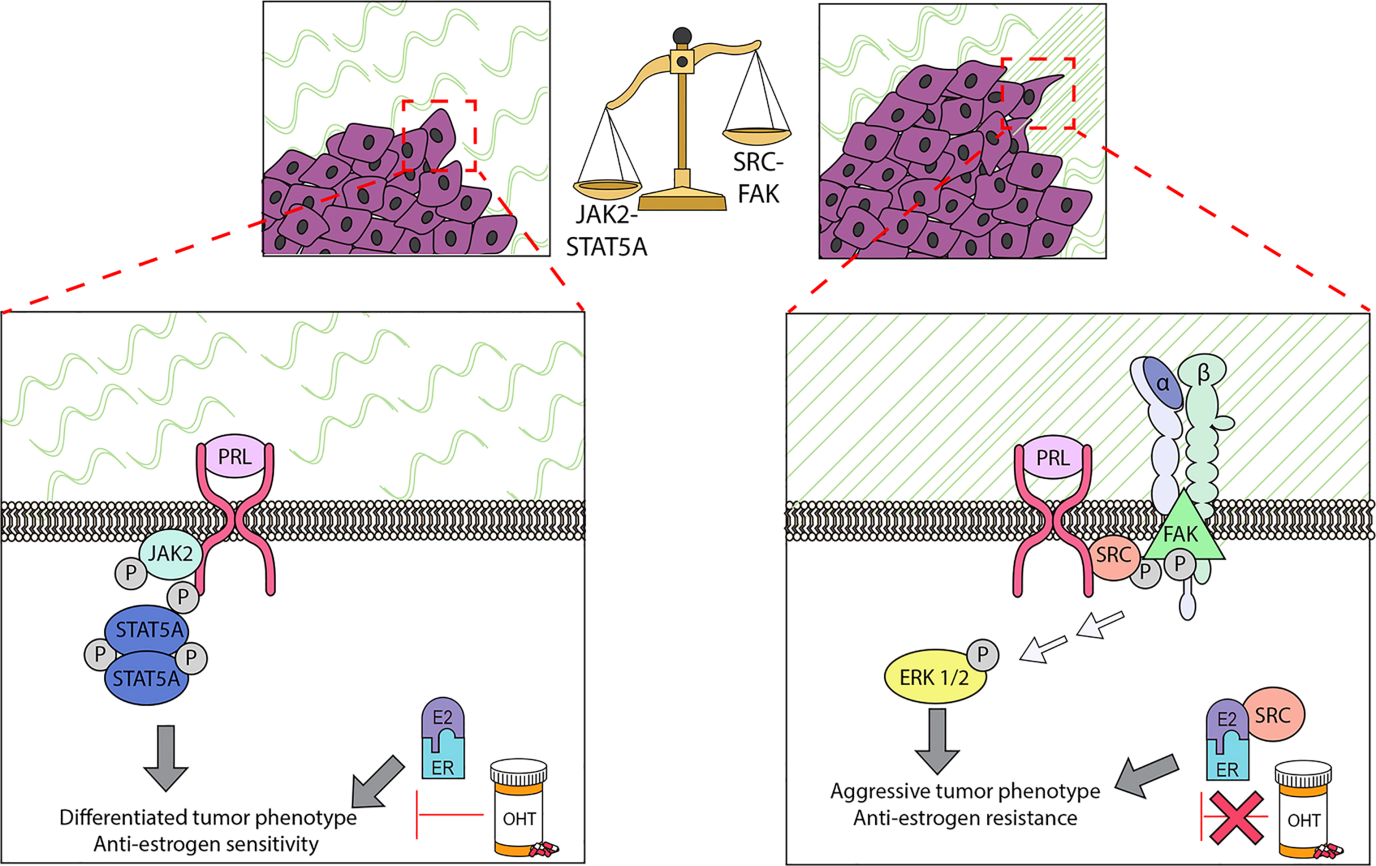
PRL can initiate multiple signaling cascades in established cancers, which can result in different biological outcomes. Determination of the repertoire of PRL signals can be modulated by multiple factors, including properties of the ECM. In ER+ cell lines, the stiffness and density of the extracellular matrix (ECM) strongly influences the balance of these signals: in stiff matrices, PRL signals are shifted away from the canonical JAK2/STAT5A pathway, and toward FAK/SFK/ERK1/2. This shift permits PRL to drive proliferation, invasion, and resistance to tamoxifen (OHT), and further remodel collagen fibers in the ECM. These experimental findings support the clinical observations that ER+ cancers with regions of aligned collagen perpendicular to the tumor boundary have a worse prognosis, and that activated STAT5A is strongly linked to more differentiated cancers and tamoxifen sensitivity. See Section 3.4 for details.

As described above, most studies have examined the outcome of the sum of all PRL-initiated signals in different breast cancer settings. However, it has long been appreciated that PRL can activate multiple signaling cascades (22, 51), with potentially different outcomes. The JAK2-STAT5A pathway mediates PRL-driven proliferation and differentiation that is essential for successful lactation (2, 154, 155), and binding of STAT5A to regulatory enhancer regions initiates chromatin remodeling, coordinating tissue specific gene expression (4, 156). (*See review by Clevenger and colleagues elsewhere in this series*). Transgenic overexpression of STAT5A leads to mammary carcinomas in the absence of other oncogenes (46). However, evidence for high STAT5A activity in clinical breast cancers has been repeatedly associated with more differentiated cancers and better prognoses [ (154, 157, 158) and references therein]. Consistently, the JAK2-STAT5A pathway also has been linked to PRL-induced pro-differentiation activities in various experimental models (53, 56, 159–161) (**Figure 2**). Of particular interest for premenopausal breast cancer, PRL-activated STAT5 suppressed a progestin-induced progenitor population in T47D breast cancer cells (162).

The highly homologous STAT5B remains a complication in these studies. In contrast to STAT5A, STAT5B is not associated with a more favorable prognosis in patients (158), and *in vitro*, STAT5B drives aggressive behavior in several models (56, 159, 161). Activities distinct from STAT5A are supported by different target genes (56, 158, 163), and divergent regulation by estrogen activity (56). Additional studies with more specific reagents are needed to resolve the roles of the STAT5 isoforms in PRL actions in breast cancer.

#### 3.4.2 Other Signaling Cascades

PRL can also initiate activation of src family kinases, and multiple additional mediators, including AKT and MAP kinases (164–168). Interestingly, the alternatively spliced PRLR isoforms with distinct intracellular domains are less able to activate the JAK2/STAT5 pathway than the well-studied “long” PRLR isoform. Indeed, heterodimerization of the “long” PRLR isoform with the “intermediate” isoform, which was recently reported to be highly expressed in a subset of TNBC (see Section 3.1), inhibits phosphorylation of STAT5, without impacting other PRL-activated signaling pathways (117). AKT and MAP kinase cascades are linked to tumor progression for many cancers (169–173). Moreover, as noted above, they can activate ERα in the absence of ligand (105, 106), with implications for therapeutic responses to anti-estrogens in ER+ breast cancers. Importantly, they are also potent sites of cooperation with other oncogenic factors, including growth factors (112, 142). Together, these observations point to the potentially divergent outcomes of different arms of PRL signals, and raise the question of determinants of the repertoire of signaling options for PRL. Clearly intrinsic differences in tumor cells themselves, including relative levels of PRLR isoforms and other signaling components play a role; different breast cancer cell lines, even of the same breast cancer subtype, exhibit different spectra of PRL activated signals *in vitro* [e.g., (137, 174)]. In addition, as discussed below, environmental factors also can powerfully modulate the balance of PRL signals.

#### 3.4.3 Features of the Extracellular Matrix Shift PRL-Initiated Signals in ER+ Tumor Cells and Alter Sensitivity To Anti-Estrogens

Accumulating data underscore the importance of the ECM in normal mammary function and tumor behavior (86, 175). A mechanically stiff matrix increases signaling through focal adhesions (176, 177). Aligned collagen fibers oriented perpendicularly to the tumor boundary have been linked to a poor prognosis, particularly in ER+ breast cancer (178). We have demonstrated that ECM structure can strongly influence the spectrum of PRL signals and PRL-estrogen crosstalk in ER+ breast cancer cells, and reciprocally, that these hormones can modify ECM structure (**Figure 2**). When well-characterized ER+ breast cancer cell lines were cultured in stiff ECM *in vitro* (MCF7 and T47D cells in 3-dimensional collagen cultures and tunable polyacrylamide substrates), PRL was less able to activate JAK2/STAT5, but more strongly activated FAK-SRC-ERK1/2, associated with increased localization of PRLR in focal adhesions (167, 179). These conditions augmented PRL-driven invasion and re-orientation of collagen fibers *in vitro* (167), and intravasation and metastasis of PRL-initiated ER+ tumors in a syngeneic model of increased COL1A1 density/stiffness *in vivo* (180). Moreover, a stiff/dense matrix enhanced PRL-estrogen crosstalk to increase invasion, reduce responsiveness to tamoxifen, and further modify ECM structure *in vitro* (181); these findings were supported using the syngeneic ER+ model above (55). Moreover, progesterone further augmented PRL induction of *MMP3* RNA in stiff matrices (107). These studies indicate that desmoplasia, a feature of the microenvironment of many tumors, can alter the repertoire of PRL-initiated signals to favor pro-tumor pathways and anti-estrogen resistance, thus illuminating one mechanism underlying the apparent disparate reports of the outcomes of PRL signals in ER+ breast cancers. Extension of these studies of the effect of ECM characteristics on PRL signals to other breast cancer subtypes may further resolve some of the apparently contradictory reports.

## 4 Summary and Future Directions

Strong epidemiologic data linking higher levels of circulating PRL to increased risk for ER+ breast cancer are supported by multiple lines of experimental evidence. Independently from ovarian steroids, PRL can modulate the epithelial hierarchy and increase progenitor populations, drive development of ductal and alveolar abnormalities, and with time, promote aggressive metastatic ER+ carcinomas. PRL engages in complex crosstalk with estrogen and progesterone, cooperating with them by multiple mechanisms, but also opposing steroid-driven differentiation. Further understanding of these interactions apart from the hormonal milieu of pregnancy will provide additional insight into the impact of PRL on increased breast cancer risk in premenopausal women and postmenopausal women treated with estrogen-progesterone MHT (107, 162, 182). As disease progresses with dysregulation of multiple pathways, intrinsic tumor cell properties and the stromal environment are likely to alter the responses of target cells to PRL and its interactions with other potential oncogenic factors. Although not well understood, the literature suggests that PRL also may act directly on multiple mammary stromal cell types including immune cell subpopulations and/or modulate their activity *via* paracrine signals, which may further increase risk. The high PRLR expression in clinical DCIS and preneoplastic structures in preclinical models is reminiscent of ER expression in many of these lesions [reviewed in (183)], and suggests a role for PRL at this early stage of the disease process.

**Figure 3:**
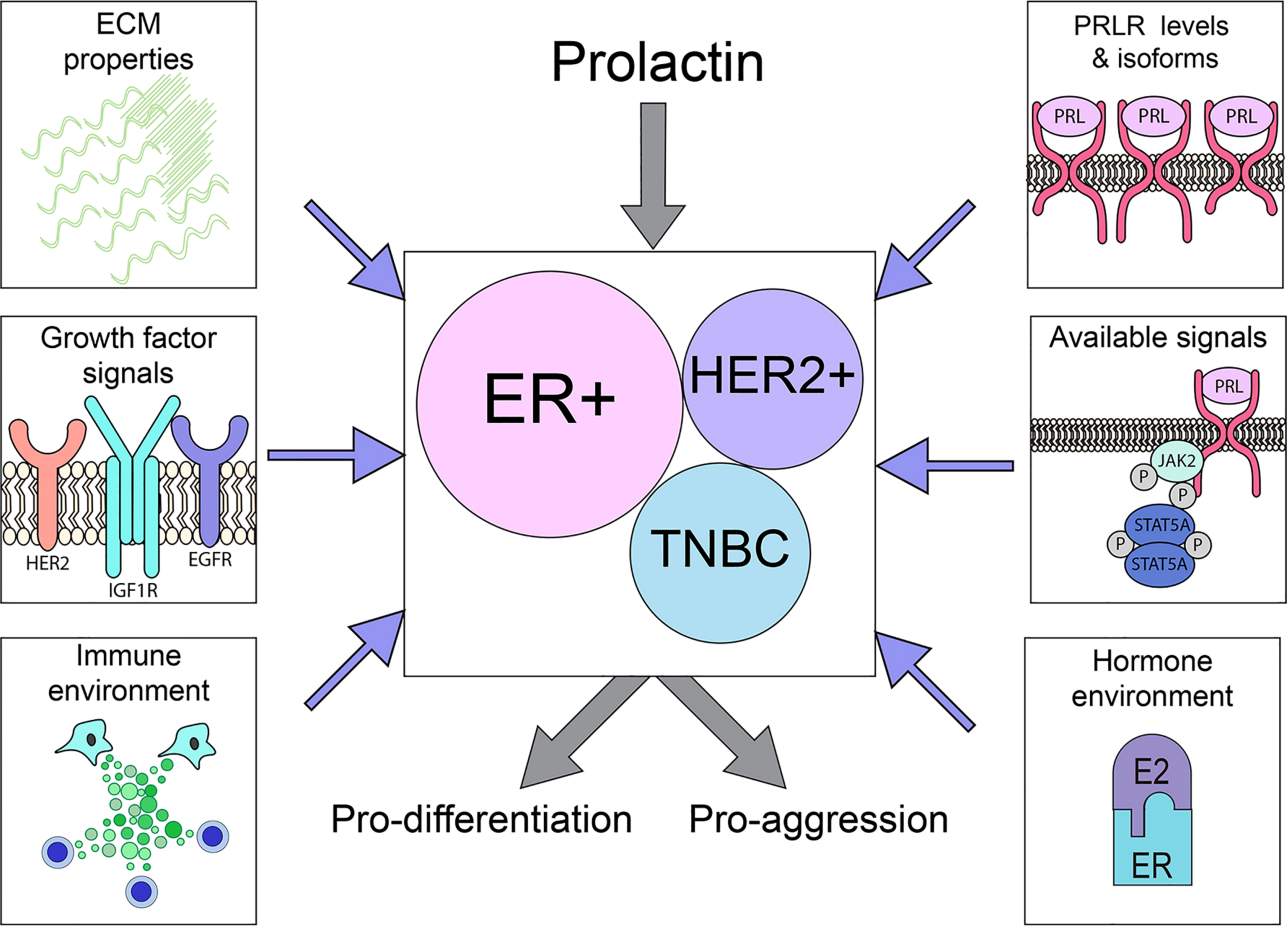
PRLR is expressed on a substantial subset of breast cancers across the major subtypes. The outcome of PRL signals may vary considerably, driving either differentiation or aggression, depending on tumor cell intrinsic properties (PRLR expression, available downstream signaling cascades) as well as extrinsic factors (ECM characteristics, hormone/growth factor milieu), as illustrated above. See Section 3 for details.

In contrast, the role(s) of PRL in the biology of established clinical breast cancers remains unclear. Although PRLR is highly expressed by many tumors across breast cancer subtypes, data from small clinical trials inhibiting PRL action are difficult to interpret, and studies of xenografts, particularly of breast cancer cell lines, are conflicting. Responses of phenotypically diverse heterogeneous cancers are complicated by different levels of PRLR isoforms with distinct signaling capabilities, selection and genomic evolution as tumors progress and respond to initial therapies, and environmental context, including site-specific responses of the metastatic niche [e.g., bone (120)], ECM properties and the steroid hormone and growth factor milieu (**Figure 3**). The emerging data support complex actions of PRL in breast cancer biology, resembling the major recognized hormonal actor in breast cancer, estrogen (184–186). This is illustrated in ER+ cancers, the breast cancer subtype in which PRL actions are currently best understood. Experimental evidence shows that PRL can activate STAT5A-driven differentiation, and maintain ERα expression, thereby facilitating anti-estrogen responsiveness (56, 128, 132, 159, 161). However, PRL can also drive proliferation and invasion, and support development of resistance to anti-estrogens (128, 181). Within the heterogeneous TNBCs, evidence indicates distinct subgroups which exhibit divergent responses to PRL (117, 122). Conflicting outcomes in a very limited number of different HER2+ breast cancer cell lines suggest similar possibilities. Together, these reports paint a more nuanced picture of PRL action in established cancers, and potential for very different outcomes depending on context. They underscore the need for additional study of PRL in diverse clinical breast cancers, changes with disease progression and therapeutic pressure, and influences of the metastatic sites.

New technologies will assist in the resolution of these issues. The NSG-Pro mouse is a powerful tool to interrogate the actions of PRL in diverse clinical breast cancers in a dynamic *in vivo* environment (128). Already providing insights into ER+ cancers, this model will help resolve some of the conflicting studies observed with experimental xenografts of a relatively small number of breast cancer cell lines of other subtypes. It will enable dissection of the mechanisms underlying observed differences, and facilitate identification of biomarkers that predict beneficial or adverse responses to PRL and/or PRL inhibitors. Pending validation of humanized mice, syngeneic mouse models continue to be essential to reveal the impact of PRL as well as other agents on inflammation and suppression of anti-tumor immunity, a critical step toward employment of the promise of immunotherapies for hormonally responsive cancers. Further, as discussed in Section 2.2.3, many other stromal cell types which sculpt the tumor microenvironment are potential PRL targets, motivating additional study in the context of breast cancers. In addition, the paucity of inhibitors to interrogate PRL actions in clinical samples and experimental models is now addressed by small molecule inhibitors (129), the technology to reduce specific PRLR isoforms *in vivo* (130), and renewed interest in PRLR neutralizing antibodies (Sections 3.1, 3.3). Development of selective inhibitors of the JAK2 and src family kinase-mediated signals of PRL, taking advantage of our understanding of the closely related growth hormone receptor, will advance these studies (142, 187, 188). Together, these approaches will unravel the complex actions of PRL, permitting a new understanding of the role of this third hormone in breast cancer, with implications for prevention and treatment.

## Author Contributions

Both LS and KO’L contributed to the original drafts and editing process. All authors have read and agreed to the published version of the manuscript.

## Funding

This work was supported by NIH R01 CA179556, P30 CA014520 (University of Wisconsin Carbone Comprehensive Cancer Center), and More for Stage IV.

## Conflict of Interest

The authors declare that the research was conducted in the absence of any commercial or financial relationships that could be construed as a potential conflict of interest.

## Publisher’s Note

All claims expressed in this article are solely those of the authors and do not necessarily represent those of their affiliated organizations, or those of the publisher, the editors and the reviewers. Any product that may be evaluated in this article, or claim that may be made by its manufacturer, is not guaranteed or endorsed by the publisher.

## Abbreviations

APC, adenomatous polyposis coli; COL1A1, collagen type I alpha 1; DCIS, ductal carcinoma in situ; ECM, extracellular matrix; ER, estrogen receptor alpha; FAK, focal adhesion kinase; GH, growth hormone; JAK2, janus kinase 2; MHT, menopausal hormone therapy; MMP, matrix metalloprotease; MMTV, mouse mammary tumor virus; NOD, non-obese diabetic; NR4A, nuclear receptor subfamily 4 group A member 1; NRL, neu-related lipocalin; NSG, NOD SCID gamma; OHT, hydroxytamoxifen; PDX, patient-derived xenograft; PGE2, prostaglandin E2; PRL, prolactin; PRLR, prolactin receptor; PTGS2, prostaglandin-endoperoxide synthase 2; RCAS, replication-competent ASLV long terminal repeat (LTR) with a splice acceptor; SCID, severe combined immunodeficiency; STAT, signal transducer and activator of transcription; TNBC, triple negative breast cancer.
